# Assessment of clinical characteristics, treatment responses, relapses, and survival in patients with thrombotic thrombocytopenic purpura undergoing therapeutic plasma exchange: A single-center experience

**DOI:** 10.12669/pjms.42.4.14962

**Published:** 2026-04

**Authors:** Ali Dogan, Ramazan Ipek

**Affiliations:** 1 Ali Dogan, MD. Hematologist, Faculty of Medicine, Hematology Department, Van Yuzuncu Yil University, Van, Turkiye; 2 Ramazan Ipek, MD. Hematologist, Faculty of Medicine, Hematology Department, Van Yuzuncu Yil University, Van, Turkiye

**Keywords:** ADAMTS13, Inhibitor, PLASMIC score, Plasma exchange, Therapeutic, Thrombotic thrombocytopenic purpura

## Abstract

**Objective:**

The present study evaluates the clinical characteristics, laboratory values, treatment responses, relapse rates, and survival outcomes in patients diagnosed with thrombotic thrombocytopenic purpura (TTP) undergoing therapeutic plasma exchange (TPE).

**Methodology:**

Included in this retrospective single-center study were 55 patients who underwent TPE with a diagnosis of TTP between 2012 to 2025. The study was conducted between October and December 2025 at Van Yuzuncu Yil University Medical Faculty Hospital. Demographic characteristics, clinical findings, laboratory results, administered therapies, complications, treatment responses, and survival data were retrieved from the hospital automation system and patient medical records.

**Results:**

The median age of the patients was 34 years, and 67.3% were female. Based on PLASMIC scores, 76.4% of the patients were classified as high risk, among whom 36.4% tested positive for an ADAMTS13 inhibitor (a disintegrin and metalloproteinase with a thrombospondin type-1 motif, member 13). The complete response rate to TPE was 75.5%, whereas the relapse rate was 23.6%, and among those who relapsed, 84.6% tested positive for the ADAMTS13 inhibitor. The median event-free survival (EFS) was 158 months, and the overall survival (OS) rate was 92.7%. The mean OS duration was determined to be 157.2 months. The median EFS was 64.8 months in those who tested positive for the ADAMTS13 inhibitor, and 158 months in those who tested negative.

**Conclusion:**

In the present study, TPE achieved high response and survival rates. High PLASMIC scores strengthen the clinical utility of the scoring system in the diagnosis of TTP. ADAMTS13 inhibitor positivity was a significant indicator of relapse risk.

## INTRODUCTION

Thrombotic thrombocytopenic purpura (TTP) is a thrombotic microangiopathy (TMA) characterized by widespread microvascular thrombus formation, marked thrombocytopenia, microangiopathic hemolytic anemia (MAHA), fever, renal insufficiency, and neurological involvement.^1^,^2^ Severe deficiencies of ADAMTS13 enzyme activity or the presence of inhibitors against this enzyme underlie the pathogenesis of TTP.^3^ The ADAMTS13 enzyme prevents excessive platelet aggregation by cleaving Von Willebrand factor (VWF) multimers into appropriately sized fragments. Decreases in ADAMTS13 enzyme activity result in inadequate cleavage of ultra-large VWF multimers, leading to microvascular thrombus formation.^3^

Etiologically, TTP is divided into two groups: congenital (cTTP) and acquired immune-mediated (iTTP).^4^ cTTP is characterized by severe ADAMTS13 deficiency resulting from ADAMTS13 gene mutations in the absence of inhibitors, while iTTP is caused by autoantibodies directed against ADAMTS13 and can be associated with various predisposing conditions such as infections, autoimmune disorders, pregnancy, neoplasms, hematopoietic stem cell transplantation, and drugs.^3^,^5^ The vast majority of adult TTP cases are immune-mediated (iTTP).

Therapeutic plasma exchange (TPE) is presently regarded as the first-line treatment for TTP, achieving response rates of approximately 80% and survival rates exceeding 90%.^3^,^6^ In addition to TPE, the use of corticosteroids, immunosuppressive therapies, and particularly rituximab, increases remission rates and decreases the frequency of relapses in iTTP cases.^5^,^7^

There have been few large-scale single-center studies reported in the literature to date. In particular, there is a paucity of large series evaluating ADAMTS13 enzyme activity and the presence of inhibitors in association with clinical characteristics, relapse, and survival rates. The present study aimed to fill this gap through an investigation of the clinical characteristics and laboratory findings, treatment responses, relapse rates, and survival outcomes in TTP patients undergoing TPE. The study also seeks to contribute to the current body of literature related to clinical practice through its investigation of potential prognostic factors.

## METHODOLOGY

Data from patients who underwent TPE due to MAHA at Van Yuzuncu Yil University Medical Faculty Hospital between January 2012 to August 2025 were reviewed for this retrospective, single-center study. The study was conducted between October and December 2025.

### Ethical approval:

The study protocol was granted approval by the Ethics Committee of our university (Approval Date: October 17^th^, 2025, decision number: 2025/10-23).

### Patient selection:

The data of 87 patients who underwent TPE due to MAHA were accessible from the hospital archive, and those with ADAMTS13 enzyme activity below the normal range were classified as having TTP. A total of 22 patients were excluded from the study, including 18 patients whose ADAMTS13 activity was within the normal range (40–130%), two who did not complete the therapy, and two who had missing data. The final study group comprised 55 patients.

### Inclusion criteria:

Included in the study were patients aged 18 and 90 years who underwent TPE with a diagnosis of TTP, and whose complete and accurate clinical, laboratory, and follow-up data were accessible from the medical records.

### Exclusion criteria:

Excluded from the study were patients with incomplete treatment records or missing response evaluation data, and those who did not comply with the treatment. Cases in which TPE was performed due to MAHA but ADAMTS13 enzyme activity was normal, cases in which the diagnosis was associated with another secondary thrombotic microangiopathy, and cases without sufficient follow up time for survival analysis were among the other exclusion criteria.

### Data collection:

Data on TPE procedures, including the number of sessions, treatment frequency, types of replacement fluids used, and plasma volume processed, were obtained from the records of the therapeutic apheresis center. Demographic characteristics such as age and sex, comorbid conditions, complications, laboratory findings, and treatment response data were retrieved from the hospital information system and medical files of the patients. Survival times were calculated from the TPE initiation date, overall treatment duration, relapse dates for those who relapsed, dates of death retrieved from the E-nabiz system, and the date of last clinical follow-up.

### Assessment of Survival, TPE Treatment Response and PLASMIC Score Data Used in the Study:

Overall Survival (OS): Overall survival was defined as the time from the initiation of treatment until death from any cause. Patients who survived were followed until the date of their last follow-up. Event-Free Survival (EFS): Event-free survival was defined as the time from the initiation of treatment to either TTP refractoriness, relapse or death from any cause, whichever occurred first. Patients who did not experience any of these events were followed until the date of their last follow-up. TPE treatment responses were classified as complete remission, partial remission, or non-response – a classification based on the clinical laboratory response criteria stated in the 2023 guidelines of the American Society for Apheresis (ASFA).^6^ The PLASMIC score is defined as: platelet count <30,000/µL, hemolysis (defined by reticulocyte count >2.5 percent, undetectable haptoglobin, or indirect bilirubin >2 mg/dL), no active cancer, no solid organ or stem cell transplant, mean corpuscular volume <90 fL, International normalized ratio <1.5, creatinine <2.0 mg/dL. The PLASMIC score was calculated by assigning one point for each applicable parameter.

### Statistical analysis:

Clinical variables and laboratory results were summarized using descriptive statistics. Categorical variables were expressed as numbers and percentages, and continuous variables as medians (minimum-maximum). Mean values were used when the median could not be calculated. The Kaplan–Meier method was used for the survival analysis, and a log-rank test for intergroup comparisons. Univariate and multivariate analyses were conducted using Cox regression models to evaluate prognostic factors. For the analysis of the difference between laboratory parameters before and after plasmapheresis, variables with normal distribution were examined with a paired samples t-test, whereas variables without normal distribution were analyzed with a Wilcoxon signed-rank test. Continuous variables were expressed as median (Q1–Q3). A p-value of less than 0.05 was considered statistically significant. All analyses were performed using SPSS Statistics (Version 22.0. IBM Corp., Armonk, NY).

## RESULTS

A total of 55 patients with TTP who underwent TPE were included in this study. The median age of the patients was 34 years (range:19–87 years) and 67.3% (n=37) were female. Among the patients, 52.7% (n=29) had no comorbid conditions. Based on PLASMIC scores at the time of diagnosis, 76.4% (n=42) were classified as high risk. ADAMTS13 inhibitor was detected in 36.4% (n=20) of the patients. The rate of complete response to TPE therapy was 75.5% (n=41). Relapse was observed in 23.6% (n=13) of the patients, and among these, ADAMTS13 inhibitor was detected in 11. Relapse occurred within the first six months in seven patients and four patients experienced more than one relapse. No deaths occurred among those who relapsed. Rituximab-based therapies were administered to 27.2% (n=15) of the patients. An evaluation of OS revealed that 92.7% (n=51) of the patients survived to the end of the follow-up period, whereas 7.3% (n=4) had died. The demographic and clinical characteristics of the patients are presented in Table-I.

**Table-I:** Demographic and clinical characteristics of TTP patients treated with TPE.

Clinical parameters	n (%) or median (min-max)
Age, years	34 (19-87)
***Gender***	
Female	37 (67.3)
Male	18 (32.7)
***Additional illness***	
Absent	26 (47.3)
Present	29 (52.7)
***PLASMİC score risk level***	
High risk	42 (76.4)
Medium risk	11 (20)
Low risk	2 (3.6)
PLASMİC score, number	6 (4-7)
Number of TPE sessions	9 (3-56)
***Frequency of TPE sessions***	
Once daily	49 (89.1)
Twice daily	6 (10.9)
***Anticoagulant used***	
Asit sitrat dextroz-A	30 (54.5)
Heparin	25 (45.5)
***Response to TPE therapy***	
Complete response	41 (74.5)
Partial response	8 (14.5)
No response	6 (10.9)
***ADAMTS13 inhibitor presence***	
Positive	20 (36.4)
Negative	35 (63.6)
***Relapse status***	
Yes	13 (23.6)
None	42 (76.4)
***Relapse in ADAMTS13 inhibitor positive patients***	
Relapse present	11 (19.8)
Relapse Absent	9 (16.2)
***Relapse in ADAMTS13 inhibitor negative patients***	
Relapse present	2 (3.6)
Relapse absent	33 (60.4)
ADAMTS13 activity (%)	6.8 (0.02-33)
ADAMTS13 inhibitor (BU/mL)	10 (2.2-80)
***Steroid and non-steroid therapies***	
Steroid administered	46 (83.63)
Rituximab	11 (20)
Rituximab+Vincristine	2 (3.6)
Rituximab+ Vincristine+ N-acetylcysteine	1 (1.8)
Rituximab+ N-acetylcysteine +Caplacizumab	1 (1.8)
Tromboprofilaksi use	47 (85.45)
***Survival status***	
Alive	51 (92.7)
Deceased	4 (7.3)

The most common symptoms at presentation were fever (21.8%) and headache (18.2%). Among the patients, 52.7% had at least one comorbid condition and 25.4% had complications associated with TPE. The patients’ symptoms, comorbid conditions, and complications are presented in Table-II. Comparison of laboratory parameters before and after TPE revealed a significant increase in platelet count and a significant decrease in LDH levels (p<0.01). Table-III presents a statistical analysis of the laboratory parameters.

**Table-II:** Patients’ presenting symptoms, comorbidities, and complications related to TPE.

Clinical parameters	n (%)
***Symptoms / Clinical findings***	
Fever	12 (21.8)
Headache	10 (18.2)
Fatigue	9 (16.4)
Altered mental status	8 (14.5)
Bleeding	7 (12.7)
Dyspnea	1 (1.8)
Other	8 (14.5)
***Comorbidities***	
None	26 (47.3)
Pregnancy / Postpartum	9 (16.36)
Diabetes mellitus	5 (9.1)
Cardiovascular disease	4 (7.3)
Chronic urticaria	4 (7.3)
Hypertension + diabetes mellitus	3 (5.5)
Hematologic malignancy	3 (5.5)
Solid malignancy	2 (3.6)
Rheumatologic disease	2 (3.6)
Cerebrovascular disease + hypertension	2 (3.6)
HIV infection	2 (3.6)
Chronic kidney disease	1 (1.8)
Intellectual disability	1 (1.8)
***Complications***	
None	41 (74.6)
Allergic reaction	4 (7.3)
Fever during plasmapheresis	3 (5.5)
Respiratory distress	2 (3.6)
Deep vein thrombosis	2 (3.6)
Catheter related complication	2 (3.6)
Pulmonary embolism	1 (1.8)

**Table-III:** Analysis of laboratory values before and after TPE application in TTP patients.

Laboratory parameters at diagnosis	Laboratory values before TPE, median (Q1–Q3)	Post-TPE laboratory values, median (Q1–Q3)	p-value
Leukocyte count (×10³/µL)	7870 (4590-10700)	8400 (5860-11600)	0.055
Platelet count (×10³/µL)	23000 (13000-37000)	200000 (117000-243000)	< 0.001
AST (U/L)	25 (17-51)	27 (21-43)	0.313
ALT (U/L)	40 (27-76)	24 (20-37)	< 0.001
Creatinine (mg/dL)	0.8 (0.63-1.41)	0.7 (0.6-0.9)	< 0.001
BUN (mg/dL)	21 (14-28)	17 (14-20)	0.029
LDH (U/L)	963 (629-1485)	250 (211-390)	< 0.001
Uric acid (mg/dL)	5.9 (4.4-6.97)	4.2 (3.8-5)	< 0.001
Total bilirubin (mg/dL)	2 (1.1-3.18)	0.6 (0.4-1)	< 0.001
Indirect bilirubin (mg/dL)	1.1 (0.47-2.5)	0.3 (0.2-0.6)	< 0.001
CRP (mg/L)	32 (14-59)	5.6 (3-13)	< 0.001
Hemoglobin (g/dL)	9.6 (7.8-11)	10 (9.1-11)	0.11

AST: Aspartate aminotransferase, ALT: Alanine aminotransferase, BUN: Blood urea nitrogen, LDH: Lactate dehydrogenase, CRP: C-reactive protein

Univariate and multivariate analyses to identify the prognostic factors for OS could not be performed because only four patients died during the follow-up period. Univariate analysis revealed high ADAMTS13 activity to be associated with prolonged EFS (HR: 0.861; 95% CI: 0.756–0.981; p=0.024), while ADAMTS13 inhibitor positivity was identified as a negative prognostic factor that significantly reduced EFS (HR: 35.052; 95% CI: 4.505–272.713; p<0.001). Multivariate analysis revealed the platelet count (HR:1,000; p=0.009), LDH (HR:1,001; p=0.047), and CRP (HR:0,927; p=0.037) values at the time of diagnosis to be independently associated with EFS. Furthermore, the presence of ADAMTS13 inhibitor was noted to be the strongest negative prognostic factor for EFS (HR: 740.604; 95% CI: 19.702–27,839.153; p<0.001). The results of the univariate and multivariate analyses investigating the prognostic factors potentially associated with EFS are presented in Table-IV.

**Table-IV:** Univariate and multivariate Cox regression analysis for prognostic factors with strong potential to influence EFS.

Clinical and laboratory parameters at diagnosis	Univariate cox analysis		Multivariate cox analysis	
	HR (95% CI)	p-value	HR (95% CI)	p-value
Age, years	0.993 (0.95-1.037)	0.739		
Gender, female*/male	1.273 (0.412-3.93)	0.675		
Number of TPE sessions, number	0.985 (0.948-1.023)	0.444		
Hemoglobin (g/dL)	0.932 (0.719-1.207)	0.592		
Platelet count (×10³/µL)	1.000 (1.000-1.000)	0.458	1.000 (1.000-1.000)	0.009
Creatinine (mg/dL)	1.132 (0.46-2.786)	0.787		
LDH (U/L)	1.000 (1.000-1.001)	0.504	1.001 (1.000-1.002)	0.047
Indirect bilirubin (mg/dL)	0.99 (0.682-1.437)	0.96		
CRP (mg/L)	0.967 (0.931-1.004)	0.077	0.927 (0.863-0.996)	0.037
ADAMTS13 activity level (%)	0.861 (0.756-0.981)	0.024		
ADAMTS13 inhibitor, present* / absent	35.052 (4.505-272.71)	<0.001	740.604 (19.702-27839.153)	<0.001
PLASMIC score, number	0.698 (0.387-1.258)	0.231		

* Reference category

**Fig.1:**
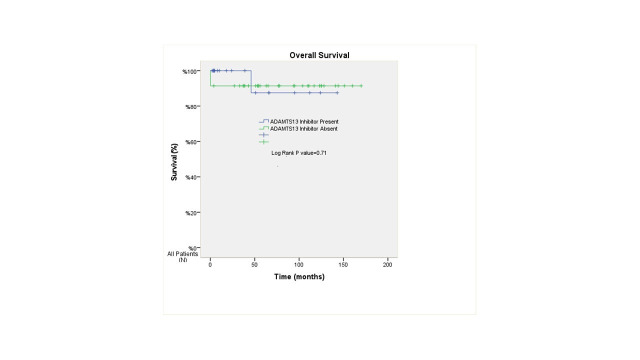
Kaplan–Meier analysis of OS in TTP patients according to ADAMTS13 inhibitor status. No statistically significant difference in OS was found between inhibitor-positive and inhibitor-negative groups (p = 0.71).

**Fig.2:**
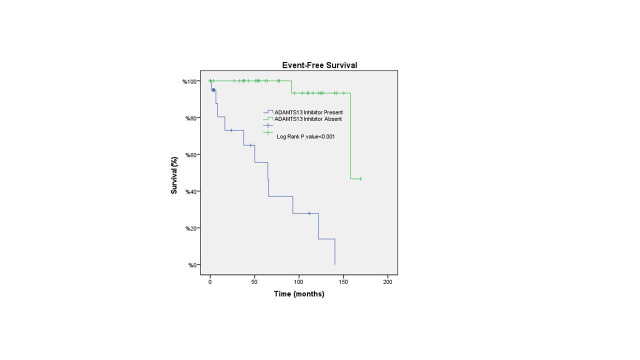
Kaplan–Meier analysis of EFS in TTP patients according to the presence of ADAMTS13 inhibitors. EFS was significantly shorter in the group with detected ADAMTS13 inhibitors compared to the group without detected inhibitors. The presence of inhibitors was identified as the strongest negative prognostic factor for EFS (p < 0.001).

**Fig.3:**
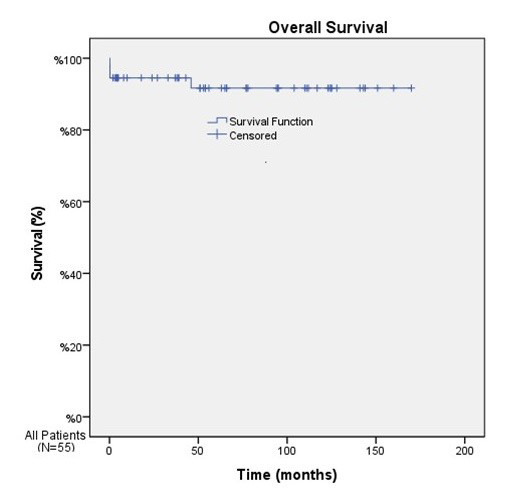
According to the Kaplan–Meier analysis of the entire cohort, the mean OS duration was calculated as 157.2 ± 6.2 months (95% CI: 145.1–169.3).

**Fig.4:**
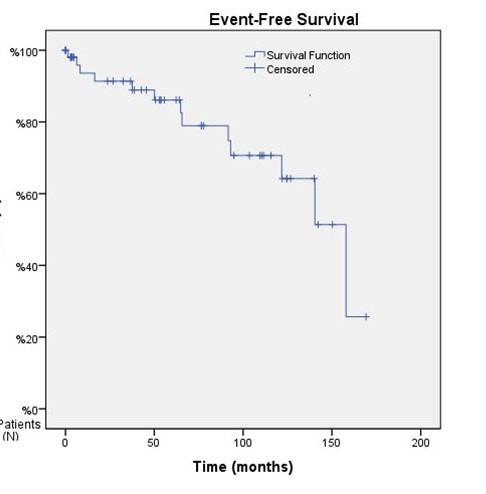
According to the Kaplan–Meier analysis of the entire cohort, the median EFS duration was calculated as 158.0 ± 18.3 months (95% CI: 122.2–193.8).

The mean OS was 130.8 months in patients with ADAMTS13 inhibitor, and 155.4 months in those without ADAMTS13 inhibitor (p=0.71) (Fig.1). Conversely, the median EFS was 64.8 months in patients with ADAMTS13 inhibitor and 158 months in patients without ADAMTS13 inhibitor (p<0.001) (Fig.2). The median OS could not be calculated because only four patients died during the follow-up period. The mean OS duration for the entire cohort was calculated as 157.2 ± 6.2 months (95% CI: 145.1–169.3) (Fig. 3), and the median EFS duration for the entire cohort was calculated as 158.0 ± 18.3 months (95% CI: 122.2–193.8) (Fig.4).

## DISCUSSION

The clinical characteristics, treatment responses, relapse rates, and survival outcomes in TTP patients undergoing TPE are evaluated in the present study. The median age was 34 years, with a predominance of female patients, consistent with previous studies reporting that iTTP predominantly affects young adults and occurs more frequently in females.^8^,^9^

Based on the PLASMIC score at the time of diagnosis, 76.4% of the patients were classified as high risk. Contemporary guidelines and studies highlight the importance of the PLASMIC score in guiding the allocation of TTP patients to TPE or corticosteroid therapy until ADAMTS13 levels become available.^6^,^10^ In a study by Karakus et al., 77.8% of 215 patients were classified as high risk for TTP based on their PLASMIC scores at the time of diagnosis.^11^ Similarly, the high rate of patients classified as high risk according to the PLASMIC score in the present study supports the increased clinical suspicion of TTP at our institution.

According to the ASFA 2023 guidelines, TPE is considered the most effective evidence-based treatment for TTP, with a Category 1 recommendation and Grade 1A level of evidence.^6^ TPE rapidly corrects the underlying pathophysiology of the condition by removing pathogenic vWF multimers from the circulation while replacing ADAMTS13 enzyme.^1^ The complete response rate was 74.5% in the present study, which concurs with the complete response rates reported in large-scale registry and cohort studies, as reflected in the ASFA 2023 guidelines.^6^ In a large series of patients diagnosed with iTTP reported by Karakus et al., the addition of corticosteroid therapy to TPE resulted in a clinical response rate of 79.9% and a mortality rate of 5.5%.^11^ The overall survival rate of 92.7% recorded at the end of the follow-up period in our study supports the efficacy of the TPE-based treatment approach.

In both univariate and multivariate analyses, ADAMTS13 inhibitor positivity was identified as a negative prognostic factor reducing EFS. In a further univariate analysis, decreased ADAMTS13 activity was found to be associated with adverse EFS outcomes. Recent studies suggest that ADAMTS13 activity and inhibitor levels are critical elements not only for diagnosis but also for predicting relapse risk and determining treatment strategies.^8^ The value of monitoring ADAMTS13 activity and inhibitor levels during the acute attack and remission periods in predicting long term relapse risk should be evaluated in prospective studies. The relapse risk for iTTP ranges from 30% to 50%.^12^ The relapse risk was 23.6% in the present study, and the majority of the relapsing patients exhibited ADAMTS13 inhibitor positivity. Rituximab is an anti-CD20 monoclonal antibody that prevents relapse by targeting B cells and inhibiting the production of anti-ADAMTS13 autoantibodies (inhibitors).^13^ Studies have shown that ADAMTS13 activity below 10%, even during remission, and the presence of ADAMTS13 autoantibodies significantly increase the risk of relapse, which can be reduced by preemptive rituximab therapy.^8^,^14^

Furthermore, studies have reported a reduced risk of relapse when rituximab is added to TPE and corticosteroid therapy during acute attacks, while others have documented the effective use of rituximab in patients with relapsing disease.^12^,^15^ In the present study, rituximab was administered to four patients who did not respond to TPE and corticosteroid therapy, along with 11 patients with relapsing disease. An evaluation of relapsing patients in the present study reveals that the administration of rituximab in patients with ADAMTS13 inhibitor positivity may be associated with a reduced relapse rate. It is important to clarify the effects of adding rituximab early to first-line therapy on relapse rates, treatment response time, and survival, particularly in high risk and ADAMTS13 inhibitor positive patients, through randomized controlled trials. In vitro studies and animal models of TTP have found that N-acetylcysteine (NAC) reduces the concentration of ultra-large VWF multimers, which suggests that NAC may be a potential treatment option through a similar mechanism in iTTP patients.^16^ In the present study, NAC was administered at 10 gram/day for three days to two patients who remained refractory to TPE, methylprednisolone, and rituximab therapies, and clinical response was achieved one of these cases.^17^ In a study by Espanol et al. involving 12 cases, it was demonstrated that NAC therapy at 150 mg/kg/day for an average of 10 days could be a beneficial and complementary treatment for acquired TTP, even resistant TTP, when used in conjunction with plasma exchange and immunosuppressive therapy.^18^ The role of NAC as a rescue therapy in refractory cases and its level of contribution to standard treatments should be clarified through comparative clinical studies.

In a large patient cohort reported by Coppo et al., the complication rate among TTP patients undergoing TPE was 24.04%.^19^ The complication rate associated with the TPE procedure in the present study was consistent with those reported in the literature. The majority of complications in our study were mild and manageable, while the rate of severe complications remained considerably low. Venous thromboembolism (VTE) developed in three patients during the TPE procedure. Two of these three patients were not receiving thromboprophylaxis. In a study by Tse et al. involving 77 patients with TTP undergoing TPE, 14% of the sample developed VTE, most commonly in the absence of pharmacological thromboprophylaxis.^20^ These findings underscore the critical importance of appropriate thromboprophylaxis in patients with TTP undergoing TPE for the prevention of thromboembolic complications.

### Strengths

A strength of the study is its analysis of the effect of ADAMTS13 inhibitor presence on relapse and survival in rare TTP cases, using long-term follow-up data. Another strength of our study is that it is based on routine clinical practice data and identifies meaningful prognostic factors in terms of EFS.

### Limitations:

The retrospective design of the study may have resulted in information gaps during the data collection process and may have introduced potential subjective biases during the evaluation of treatment responses. In the contemporary treatment era of TTP, the low mortality rate resulted in only four deaths during the follow-up period, and so potential prognostic factors associated with OS could not be evaluated. Another limitation of the study is that ADAMTS13 genetic mutation analysis was not performed.

## CONCLUSION

The present study achieved high rate of hematological response and high rate of overall survival in patients with TTP undergoing TPE. Thromboprophylaxis should be administered during TPE unless contraindicated. The present study demonstrated that ADAMTS13 inhibitor positivity has a significant negative prognostic impact on both EFS and relapse. In patients with a high PLASMIC score at the time of diagnosis, markedly reduced ADAMTS13 activity and inhibitor positivity, the early initiation of TPE and concurrent corticosteroid therapy, and the administration of rituximab in appropriate patients, constitutes the basis of the treatment strategy in accordance with the current guidelines.
